# Supplementary material for: Modeling material transport regulation and traffic jam in neurons using PDE-constrained optimization
Source: Sci Rep. 2022 Mar 10;12:3902. doi: 10.1038/s41598-022-07861-6 (PMC8913697; doi:10.1038/s41598-022-07861-6)
Supplement: Supplementary file 1 — Supplementary Information. [file 41598_2022_7861_MOESM1_ESM.pdf]

Supplementary Information
Modeling material transport regulation and traffic jam in neurons using PDE-constrained optimization

Angran Li and Yongjie Jessica Zhang

Supplementary Figures

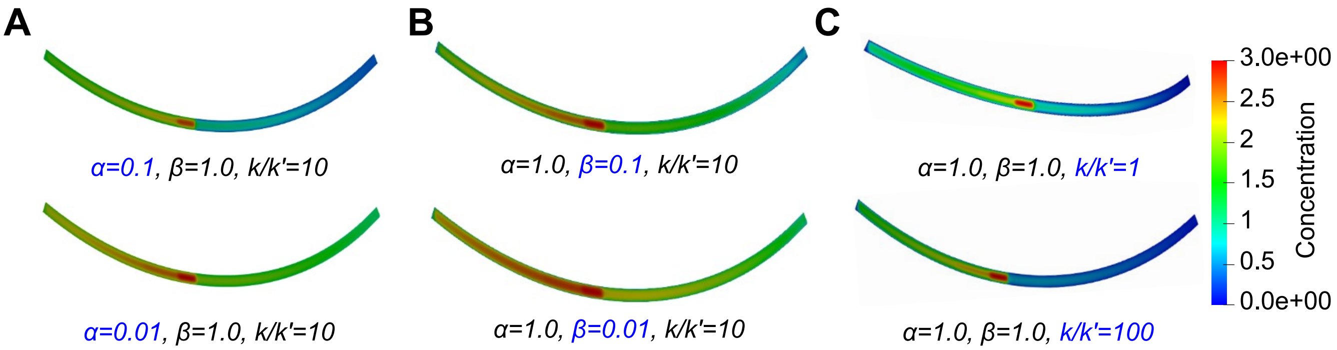

Figure S1. Parameter analysis in the single pipe geometry. The distribution of concentration affected by different settings of (A) penalty parameter α for the cost to control high concentration gradient; (B) penalty parameter β for the cost of control force; and (C) the ratio between attachment rate k and detachment rate k'. Unit for color bars: $mol/\mu m^3$.

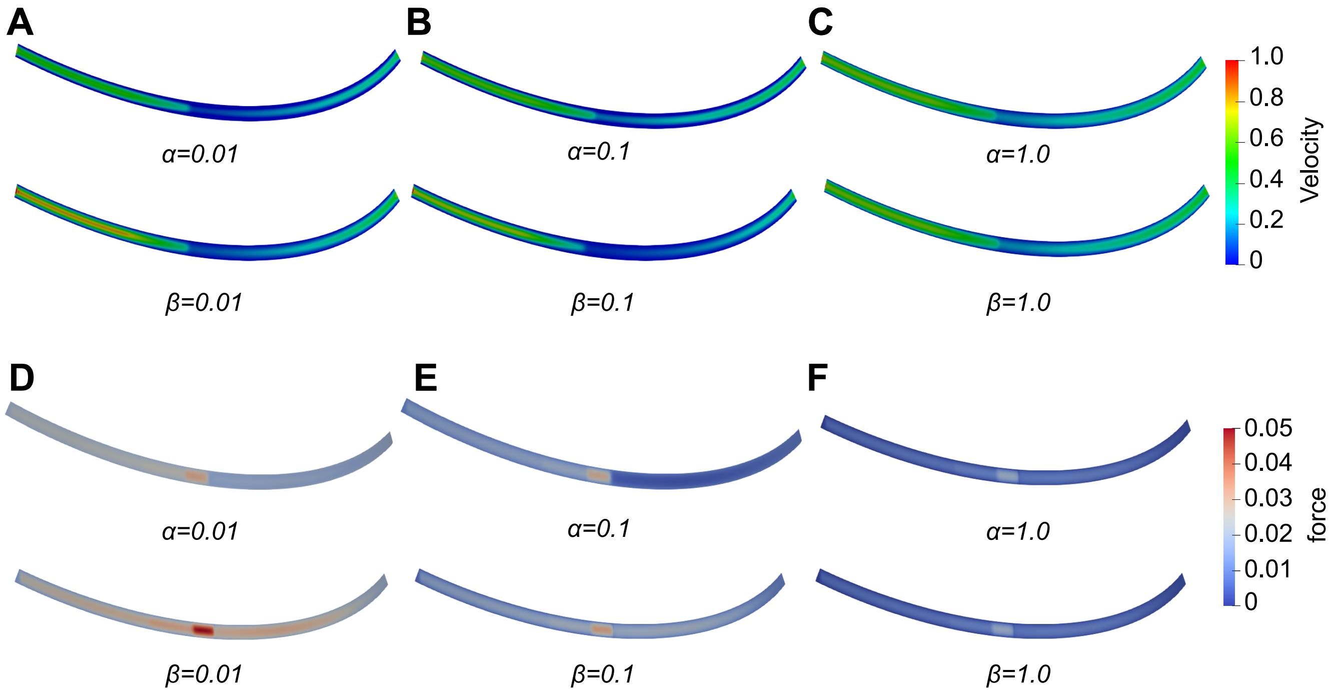

Figure S2. Simulation results in the single pipe geometry with different settings of penalty parameter α and β. (A-C) Computed velocity field. (D-F) The distribution of control force. Unit for color bars: Velocity: $\mu m/s$; Control force: $\mu m/s^2$.

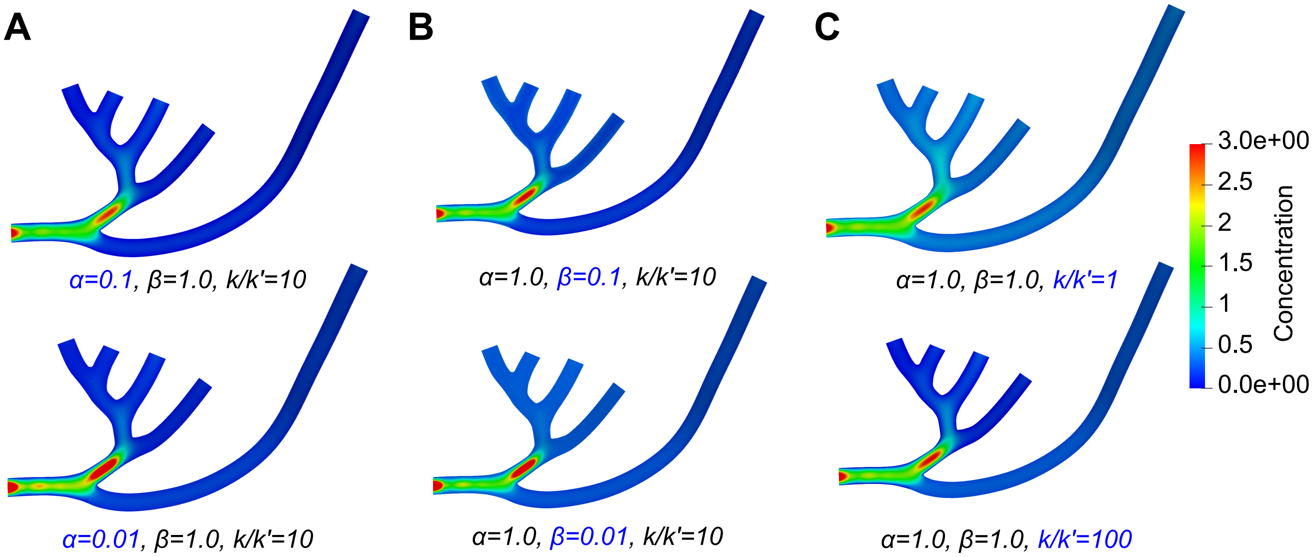

Figure S3. Parameter analysis in the neuron tree extracted from NMO_54504. The distribution of concentration affected by different settings of (A) penalty parameter α for the cost to control high concentration gradient; (B) penalty parameter β for the cost of control force; and (C) the ratio between attachment rate k and detachment rate k'. Unit for color bars: $mol/\mu m^3$.

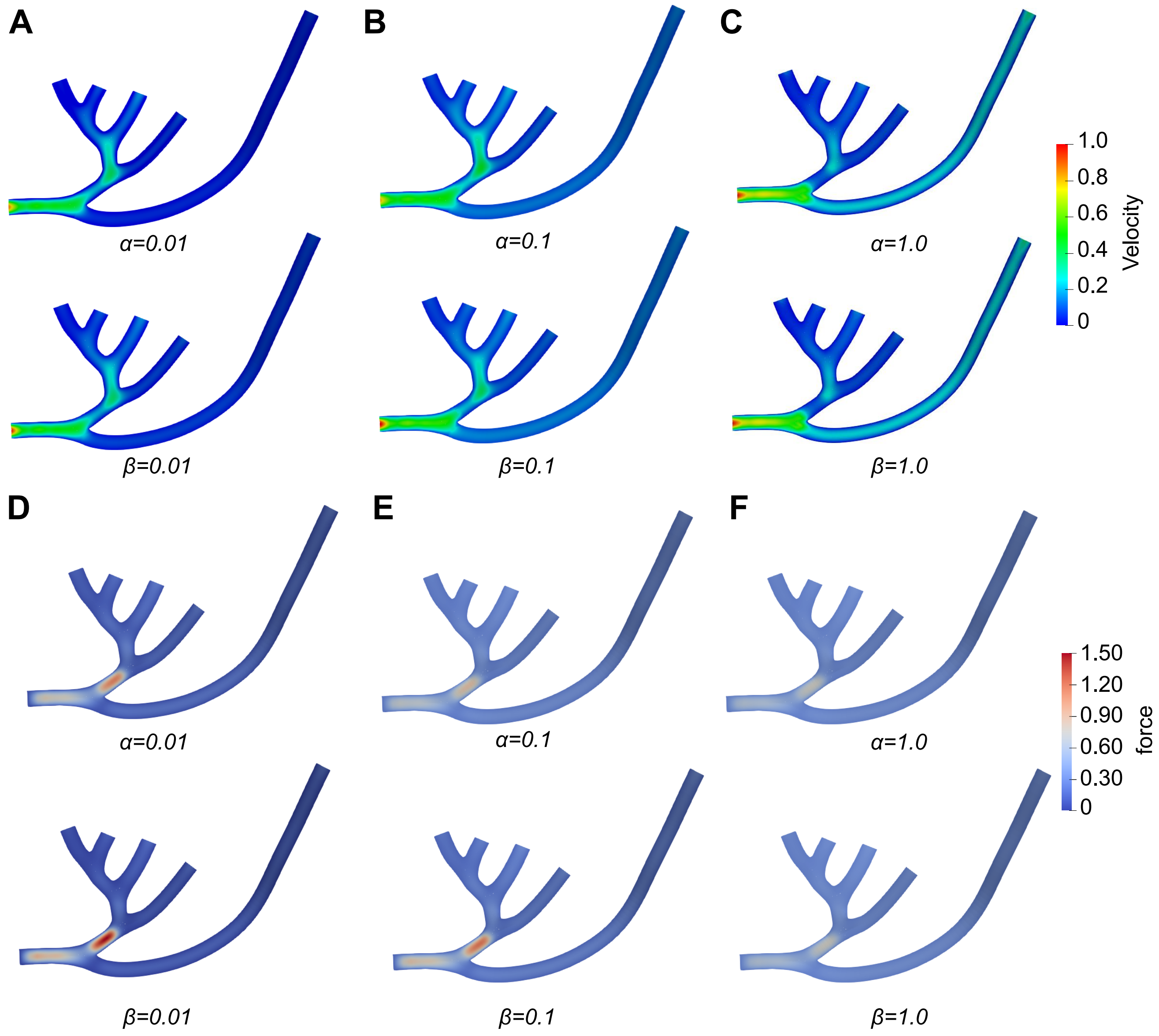

Figure S4. Simulation results in the neuron tree extracted from NMO_54504 with different settings of penalty parameter α and β. (A-C) Computed velocity field. (D-F) The distribution of control force. Unit for color bars: Velocity: $\mu m/s$; Control force: $\mu m/s^2$.

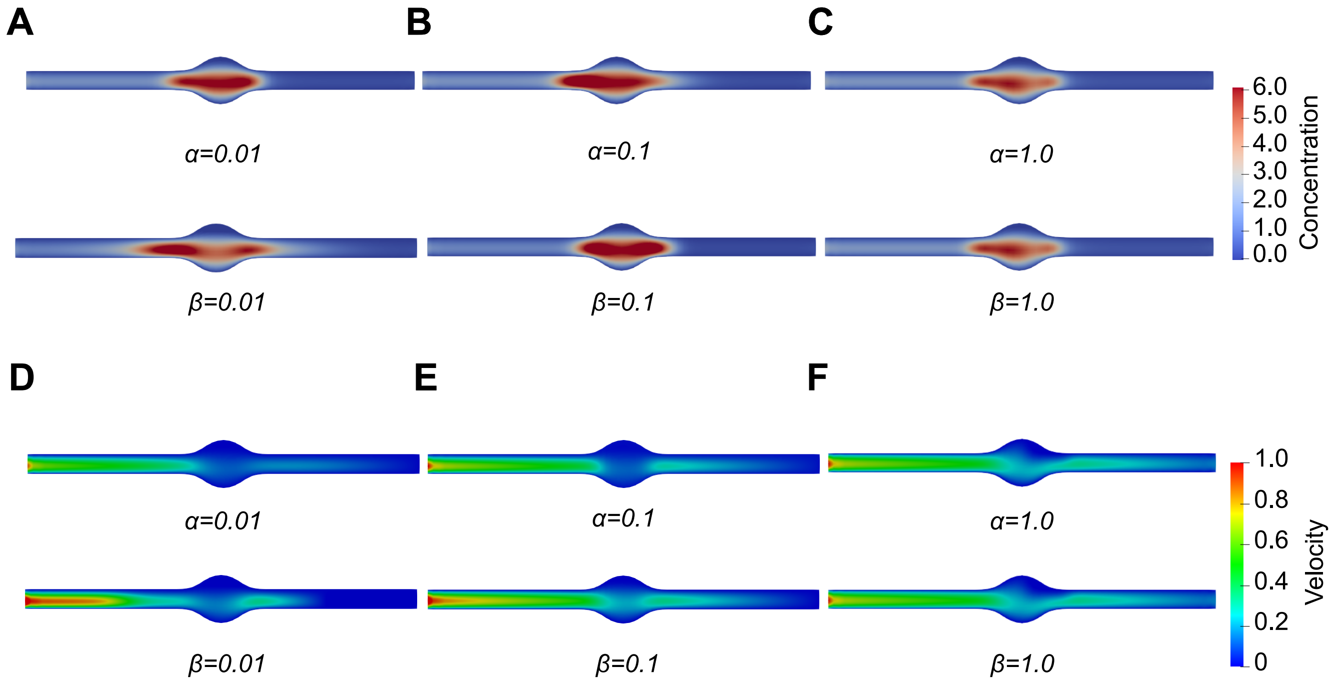

Figure S5. Simulation results in the pipe with swelling geometry with different settings of penalty parameter α and β. (A-C) Material distribution. (D-F) Computed velocity field. Unit for color bars: Concentration: $mol/\mu m^3$; Velocity: $\mu m/s$.

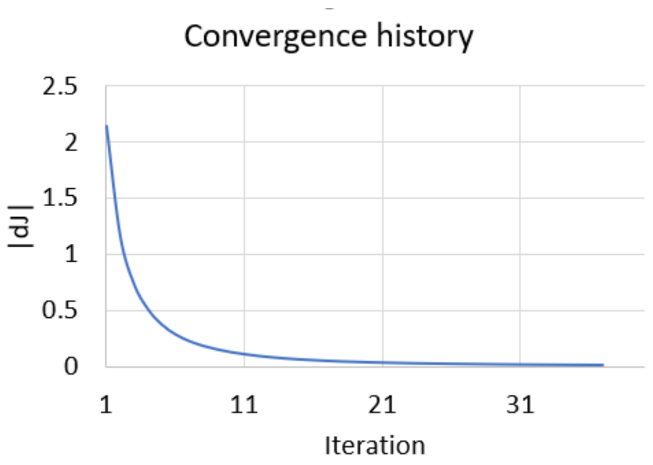

Figure S6. The convergence history of the simulation in single pipe geometry.
